# Supplementary figures and images for: Psychometric properties of a screening tool for autism in the community—The Indian Autism Screening Questionnaire (IASQ)
Source: PLoS One. 2021 Apr 22;16(4):e0249970. doi: 10.1371/journal.pone.0249970 (PMC8062015; doi:10.1371/journal.pone.0249970)

S1 Fig.

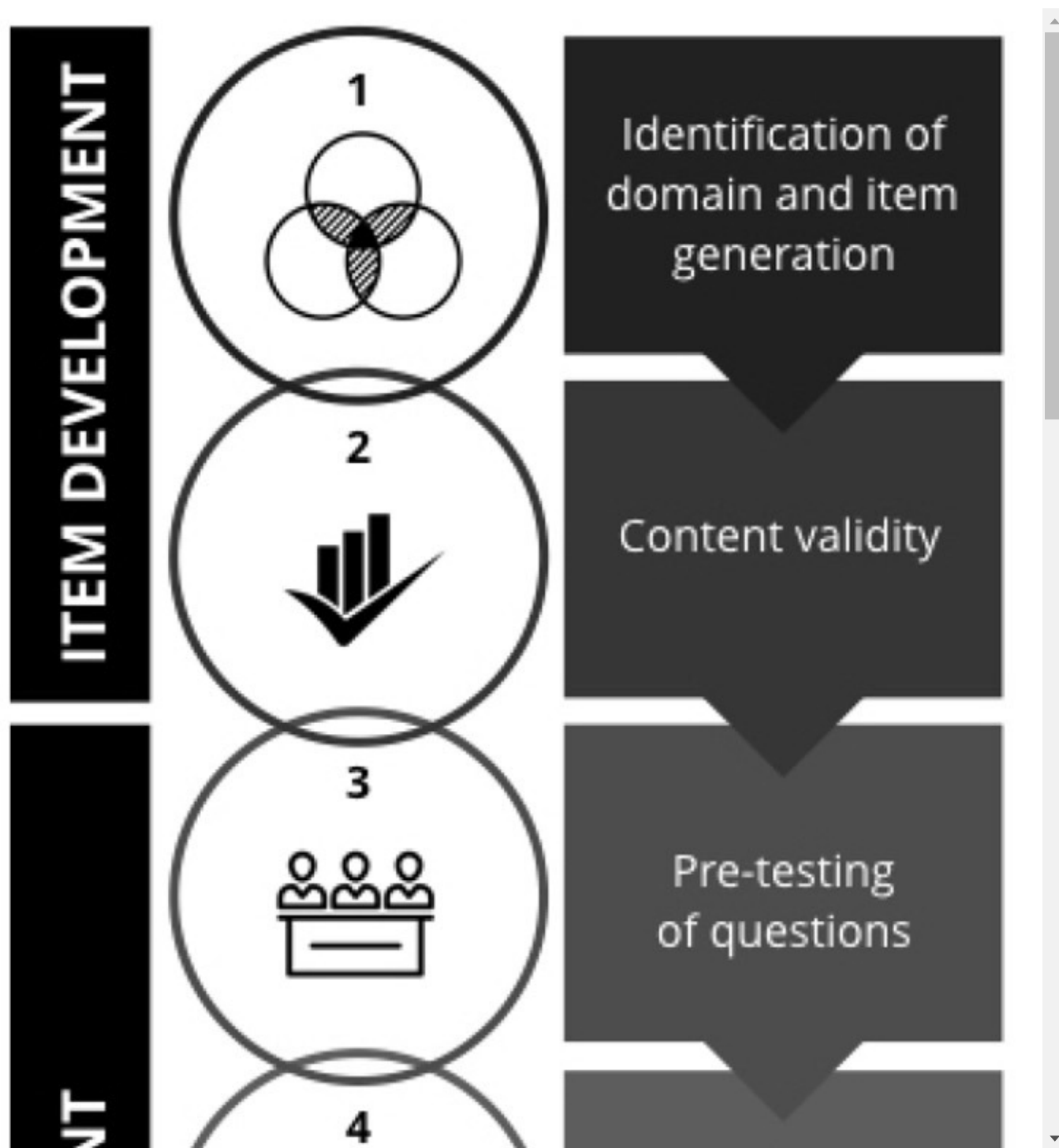

An overview of the three phases and nine steps of scale development and validation.

Supplement: S1 Fig — (PDF) [file pone.0249970.s001.pdf]
