# Supplementary material for: Psychometric properties of a screening tool for autism in the community—The Indian Autism Screening Questionnaire (IASQ)
Source: PLoS One. 2021 Apr 22;16(4):e0249970. doi: 10.1371/journal.pone.0249970 (PMC8062015; doi:10.1371/journal.pone.0249970)
Supplement: S2 Fig — (DOCX) [file pone.0249970.s002.docx]

**S2 Fig. Age-wise sensitivity and specificity of IASQ**
