# Supplementary material for: Psychometric properties of a screening tool for autism in the community—The Indian Autism Screening Questionnaire (IASQ)
Source: PLoS One. 2021 Apr 22;16(4):e0249970. doi: 10.1371/journal.pone.0249970 (PMC8062015; doi:10.1371/journal.pone.0249970)
Supplement: S1 Table — (DOCX) [file pone.0249970.s003.docx]

S1 Table. Age-wise distribution of True positive, True negative, False positive and False negative cases with Sensitivity and Specificity at cut off score of 1:

| Age group  (in years) | 3-5(n=49) | 6-8(n=28) | 9-11(n=29) | 12-14(n=22) | 15-18(n=17) |
| --- | --- | --- | --- | --- | --- |
| True Positive(N) | 33 | 23 | 12 | 12 | 9 |
| False Positive(N) | 6 | 0 | 9 | 2 | 4 |
| True Negative(N) | 10 | 5 | 8 | 8 | 3 |
| False Negative(N) | 0 | 0 | 0 | 0 | 1 |
| Sensitivity | 100% | 100% | 100% | 100% | 90% |
| Specificity | 63% | 100% | 47% | 80% | 43% |
