# Supplementary material for: Psychometric properties of a screening tool for autism in the community—The Indian Autism Screening Questionnaire (IASQ)
Source: PLoS One. 2021 Apr 22;16(4):e0249970. doi: 10.1371/journal.pone.0249970 (PMC8062015; doi:10.1371/journal.pone.0249970)
Supplement: S2 Table — (DOCX) [file pone.0249970.s004.docx]

**S2 Table. Concordance of IASQ diagnoses at various cutoff points with clinical diagnoses of autism and non-autism participants.**

| Cutoff | Autism/Not autism Participants at different cut offs of IASQ  N=145 | Cronbach alpha | p-value |
| --- | --- | --- | --- |
| 1 | 110/35 | 0.819 | <0.0001 |
| 2 | 104/41 | 0.857 | <0.0001 |
| 3 | 96/49 | 0.874 | <0.0001 |
| 4 | 92/53 | 0.856 | <0.0001 |
| 5 | 83/62 | 0.795 | <0.0001 |
| 6 | 76/69 | 0.799 | <0.0001 |
| 7 | 62/83 | 0.710 | <0.0001 |
| 8 | 43/102 | 0.666 | <0.0001 |
| 9 | 13/132 | 0.350 | 0.180 |
| 10 | 4/141 | 0.146 | 0.500 |
