# Supplementary material for: Psychometric properties of a screening tool for autism in the community—The Indian Autism Screening Questionnaire (IASQ)
Source: PLoS One. 2021 Apr 22;16(4):e0249970. doi: 10.1371/journal.pone.0249970 (PMC8062015; doi:10.1371/journal.pone.0249970)
Supplement: S2 File — (PDF) [file pone.0249970.s006.pdf]

भारतीय ऑटिज्म जांच प्रश्नावली (IASQ)

INDIAN AUTSIM SCREENING
QUESTIONARE (IASQ)

आईडी नंबर/ ID No   परीक्षक/ Examiner.

जन्म की तारीख/ Date of birth.   आयु/ Age/लिंग/ Sex.   निदान/Diagnosis.

कृपया सही प्रतिक्रिया पर टिक करें/ Please tick the correct response.

क्या आपका बच्चा / बच्ची/ Does your child

Sr. No.	Question	हाँ Yes	नही No
1	आंखों से आँखे कम मिलाता / ती है?<br>Have poor eye contact?		
2	सामाजिक स्थिति में मुस्कुराहट की कमी है?<br>Lack social smile?		
3	अलग–थलग रहता / ती है?<br>Remain aloof?		
4	लोगों से स्वयम् बढ़कर सम्पर्क नहीं करता / ती है?<br>Not reach out to others?		
5	अकेले और दोहराने वाले खेल खेलता / ती है?<br>Engage in solitary and repetitive play activities?		
6	अपने साथ के बच्चों से सम्बध नहीं बनाए रख पाता / ती है?<br>Not maintain peer relationships?		
7	लोगों के साथ न ही बातचीत की शुरूवात, और न ही उसे बनाए रख पाता / पाती है?<br>Is unable to initiate or sustain conversation with others?		
8	एक ही प्रकार की शारीरिक क्रियाओं को बार–बार दोहराता / ती है?<br>Engage in stereotyped and repetitive motor mannerisms?		
9	निर्जीव वस्तुओं से लगाव दिखाता / ती है?<br>Show attachment to inanimate objects?		
10	वस्तुओं या व्यक्तियों को, असामान्य रूप से सूंघकर, छूकर, या चखकर देखता / ती है?<br>Respond to objects/people unusually by smelling, touching or tasting?		

कुल (हाँ) / Total(Yes)________   **हस्ताक्षर / Signature_____________**

कुल (नही) / Total(No)________   **तारीख / Date__________________**

	Supplementary and Explanatory Questions to IASQ	IASQ के लिए पूरक और व्याख्यात्मक प्रश्न
1.	***Have poor eye contact?***	आंखों के संपर्क में कमी
	• *Does the child look at you when you call him/her?*<br>• *While asking something does he/she look at you?*<br>• *Does the child get fussy when forced to look at people?*	• क्या बुलाने पर बच्चा/बच्ची आपकी तरफ देखता/देखती है?<br>• कुछ पूछते समय क्या वह आपकी तरफ देखता/देखती है?<br>• बच्चे को बुला के जांच करिए क्या वह आपकी तरफ देखता/देखती है, आप माता–पिता को भी बच्चा/बच्ची को बुलाने को कहकर उसकी आंख का संपर्क जांच सकते हैं।<br>• क्या लोगों से आंख मिलाने के लिए जबरदस्ती करने पर वह चिढ़ जाता/जाती है?<br>• अगर बच्चा/बच्ची आंख मिलाता/मिलाती है तो कितनी बार तथा कितनी देर के लिए संपर्क बनाए रखता है, इसकी जांच कीजिए।
2.	***Lack social smile?***	सामाजिक मुस्कान में कमी
	• *Does the child smile in response to familiar people (Grandparents, relatives/friends)?*<br>• *Does the child smile when someone greets him/her?*<br>• *Does he/she smile when father/mother returns home in the evening?*<br>• *Does he/she greet others by smiling?*	• क्या बच्चा/बच्ची परिचित लोगों को प्रतिक्रिया देते समय मुस्कुराता/मुस्कुराती है? (दादा–दादी, संबंधी, मित्र)<br>• क्या किसी के अभिवादन करने पर बच्चा/बच्ची मुस्कुराता/मुस्कराती है?<br>• जब माता/पिता शाम को घर लौटते हैं तो क्या वह मुस्कुराता है?<br>• क्या वह औरों का मुस्कुराहट के साथ अभिवादन करता/करती है?
3.	***Remain aloof?***	अलग–थलग रहना

	• *Does the child prefer to be alone rather in a group?*<br>• *Does the child like playing with a group of children?*<br>• *Does the child want parents' presence while playing or doing any activity?*<br>• *Does the child feel comfortable being in a group?*<br>• *If he /she is engaged in group activity how does he /she react? Does he/she get irritated or fussy?*<br>• *Does he/she ignore the presence of family members?*	• क्या बच्चा/बच्ची समूह के बजाय अकेले रहना पसंद करता/करती है?<br>• क्या बच्चा/बच्ची, बच्चों के समूह के साथ खेलना पसंद करता/करती है?<br>• क्या बच्चा/बच्ची खेलते या कोई अन्य कार्य करते समय, माता–पिता की उपस्थिति चाहता/चाहती है?<br>• क्या बच्चा समूह में सहज महसूस करता है?<br>• किसी सामूहिक गतिविधि में शामिल करने पर वह किस तरह की प्रतिक्रिया देता/देती है? (क्या वह गुस्सा करता/करती है या चिढ़ जाता/जाती है)?<br>• क्या वह परिवार के सदस्यों की उपस्थिति को अनदेखा करता है/करती है?
4.	***Not reach out to others?***	लोगों से स्वयं बढ़कर संपर्क नहीं करना
	• *Does he/she like being with family members? (Parents, siblings, grandparents)*<br>• *Does he/she like playing with his/her siblings/friends?*<br>• *Does he/she like going out with family members?*<br>• *Does he/she run and hug parents/grandparents/close relatives when they return after a break?*<br>• *Is he/she attached to only one family member and ignores others' presence?*	• क्या वह परिवार के सदस्यों के साथ रहना पसंद करता/करती है? (माता–पिता, भाई–बहन, दादा–दादी)<br>• क्या वह अपने भाई–बहनों/दोस्तों के साथ पसंद करता/करती हैं।<br>• क्या वह परिवार के सदस्यों के साथ बाहर जाना पसंद करता/करती है?<br>• क्या जब उसके माता–पिता /दादा–दादी /नजदीकी रिश्तेदार कुछ समय बाद वापिस आते हैं तो उन्हें दौड़कर गले लगाता/लगाती है?<br>• क्या वह केवल परिवार के एक ही सदस्य से जुड़ा हुआ है और दूसरों की उपस्थिति को अनदेखा करता है?
5.	***Engage in solitary and repetitive play activities?***	अकेले और दोहराने वाले खेल खेलना
	• *Does the child prefer playing the same game again and again for long duration? (Like playing with crayons/cars/ beads/ strings).*<br>• *If the child's play is interrupted how does he/she react*	• क्या बच्चा लंबी अवधि के लिए बार–बार एक ही खेल खेलना पसंद करता है? (जैसे रंगों/कारों/मोतियों/तारों के साथ खेलना पसंद करना)<br>• बच्चे के खेल में बाधा आने पर वह किस प्रकार प्रतिक्रिया करता/करती है?

	• *If undisturbed how long does he/she continue the same activity? Does he/she have any fixated interests? (Like playing by keeping the cars in a line or stacking things). When interrupted how does he/she react?*	• बच्चे के खेल में रुकावट न करने पर वह कितने समय तक वही खेल खेलना जारी रखता/रखती है?<br>• क्या उसका कोई निश्चित लगाव है? (जैसे कारों को लाइन में रखना या चीजों का ढ़ेर बनाकर खेलना)<br>• बाधा लाने पर वह किस तरह प्रतिक्रिया देता/देती है?
6.	***Not maintain peer relationships?***	समान बच्चों से संबंध न बनाए रखना
	• *Does the child have friends?*<br>• *Does he/she know the names of his/her friends?*<br>• *Does he/she prefer playing with any particular child?*<br>• *Does he/she express the wish to play with his/her friends?*<br>• *Does he/she like attending his/her friend's birthday parties?*<br>• *Does he/she share his/her experiences with his/her friends?*<br>• *Does he/she feel close to any particular child?*	• क्या बच्चे के दोस्त हैं?<br>• क्या वह अपने दोस्तों के नाम जानता है/जानती है?<br>• क्या वह किसी खास बच्चे के साथ खेलना पसंद करता है/करती है?<br>• क्या वह अपने दोस्तों के साथ खेलने की इच्छा व्यक्त करता है/करती है?<br>• क्या वह अपने दोस्त के जन्मदिन की पार्टियों में भाग लेना पसंद करता है/करती है?<br>• क्या वह अपने दोस्तों के साथ अपने अनुभव साक्षा करता है/करती है?<br>• क्या वह किसी खास बच्चें के करीब महसूस करता है?
7.	***Not initiate or sustain conversations with others?***	दूसरों के साथ बातचीत शुरु करने और बनाए रखने में असमर्थ
	• *Does the child interact on his/her own with others?*<br>• *Does the child ask questions?*<br>• *Can the child sustain conversation with others (family members, teachers, and friends)?*<br>• *If yes, check the frequency and duration.*<br>• *Does he/she respond mostly by nodding his/her head or by saying yes or no?*	• क्या बच्चा दूसरों के साथ अपनी खुद की बातचीत करता है/करती है?<br>• क्या बच्चा सवाल पूछता है?<br>• क्या बच्चा दूसरों के साथ बातचीत जारी रख सकता है? परिवार के सदस्यों, शिक्षकों, दोस्तों।<br>• क्या वह अपना सिर हिलाकर या हां/ना कहकर ज्यादातर प्रतिक्रिया देता है/देती है?

8.	***Engage in stereotyped and repetitive motor mannerisms?***	बार–बार और दोहरावदार तरीके में व्यस्त रहना
	• *Does he/she engage in repetitive behaviours like hand flapping, head nodding, finger wiggling and body rocking?*<br>• *Ask if the child uses any object while engaging in repetitive activities (spinning objects).*	• क्या वह हाथ हिलाते रहना, सिर हिलाना, उंगली लड़खड़ाना और शरीर को हिलाना जैसे दोहराए जाने वाले व्यवहारों में शामिल रहता है/रहती है?<br>• कभी–कभी जांचें कि क्या बच्चा दोहराए जाने वाली गतिविधियों में शामिल होने के दौरान किसी भी वस्तु का उपयोग करता है। (घुमाने वाली वस्तुएं)
9.	***Show attachment to inanimate objects?***	नजीव वस्तुओं के प्रति लगाव दिखाना
	• *Does the child play with strings, threads/ wires?*<br>• *Does the child want to keep any object/toy with him/herself throughout the day?*<br>• *If parent tries to take it away how does he/she react?*	• क्या बच्चा रस्सी, धागे/तारों से खेलता है?<br>• क्या बच्चा किसी वस्तु/खिलौने को दिनभर अपने साथ रखना चाहता है?<br>• अगर माता–पिता इसे दूर करने की कोशिश करते हैं, तो वह कैसे प्रतिक्रिया देता है/देती है?
10.	***Respond to objects/people unusually by smelling, touching or tasting?***	वस्तुओं/लोगों को अजीब तरह से महक, स्पर्श या चखने से प्रतिक्रिया करना
	• *Does the child put everything in his/her mouth? (even inedible objects-toys, pens, pencils, crayons)*<br>• *Does the child like to smell things? (Dresses, hair, people)*<br>• *Does the child touch/hug other people?*<br>• *Does he/she express dislike when someone tries to touch him/her?*<br>• *Does the child like to eat food of a particular taste?*	• क्या बच्चा अपने मुंह में सब कुछ डालता है? (अखाद्य वस्तुएं–खिलौने, पेन, पेंसिल, रंग)<br>• क्या बच्चा चीजों को सूघंना पसंद करता है? (कपड़े, बाल, लोग)<br>• क्या बच्चा अन्य लोगों को छूता है/गले लगाता है?<br>• जब कोई उसे छूने की कोशिश करता है तो वह नापसंद व्यक्त करता है?<br>• क्या बच्चा केवल विशेष स्वाद का खाना खाना पसंद करता है?
