# Supplementary material for: Psychometric properties of a screening tool for autism in the community—The Indian Autism Screening Questionnaire (IASQ)
Source: PLoS One. 2021 Apr 22;16(4):e0249970. doi: 10.1371/journal.pone.0249970 (PMC8062015; doi:10.1371/journal.pone.0249970)
Supplement: Attachment — Submitted filename: iasq revised ltr editor ans reviewers 11032021.doc [file pone.0249970.s008.doc]

Dr. Smita N. Deshpande, MD, DPM

Professor,

Dept. of Psychiatry,

Centre of Excellence in Mental Health,

Atal Bihari Vajpayee Institute of Medical Sciences

& Dr Ram Manohar Lohia Hospital,

Banga Bandhu Sheikh Mujib Road,

New Delhi 110001

March 11, 2021

To,

Dr. Emily Chenette

Editor –in-Chief

PLOS ONE.

Attn.: Dr. Robert Didden

Ref.: Revised submission of our manuscript number PONE-D-20-39733: “ Psychometric properties of a screening tool for autism in the community- the Indian Autism Screening Questionnaire (IASQ)”


Dear Editors,

Thank you for getting our manuscript reviewed. The reviewers’ comments are thoughtful and very encouraging, as they understood the need for an instrument for community screening for autism in LMIC.

We are resubmitting our modified manuscript as per the Academic Editor’s and Respected Reviewers’ comments.

We have no legal or ethical issues for uploading anonymized data of our study, hence we have uploaded our study data along with the revised manuscript. We have deleted the subheadings in the introduction section and added the research question at the end of the introduction and methods. We have uploaded the response to reviewers, marked and unmarked copies of the modified manuscript. There is no change in financial disclosure. Figure files are also uploaded according to PLOS One format.

We hope this modified manuscript is according to the journal’s expectations and will be accepted for publication.

Thank you for an early response and acceptance if possible!

Sincerely,


Smita N Deshpande

**Reply to comments**

PONE-D-20-39733: “ Psychometric properties of a screening tool for autism in the community- the Indian Autism Screening Questionnaire (IASQ)”

**Journal Requirements:**

Please review your reference list to ensure that it is complete and correct. If you have cited papers that have been retracted, please include the rationale for doing so in the manuscript text, or remove these references and replace them with relevant current references. Any changes to the reference list should be mentioned in the rebuttal letter that accompanies your revised manuscript. If you need to cite a retracted article, indicate the article’s retracted status in the References list and also include a citation and full reference for the retraction notice.

When submitting your revision, we need you to address these additional requirements.

 1. Please ensure that your manuscript meets PLOS ONE's style requirements, including those for file naming. The PLOS ONE style templates can be found at

https://journals.plos.org/plosone/s/file?id=wjVg/PLOSOne_formatting_sample_main_body.pdf and

[https://journals.plos.org/plosone/s/file?id=ba62/PLOSOne_formatting_sample_title_authors_affiliations.pdf](about:blank)

*Reply1: The revised manuscript follows all PLOS One style requirements.*

Query 2: Please include a copy of the validated questionnaire in the original language, as Supporting Information, or include a citation if it has been published previously.

*Reply: Thank you for this suggestion. We have included the bilingual IASQ questionnaire as appendix.*

Query 3. Please discuss how the original Indian Scale for Assessment of Autism was validated prior to use in India.

*Reply 3: We have added the following text in the introduction section (see below for the entire paragraph on the ISAA):*

The Indian Scale for Assessment of Autism (ISAA) was developed by the Government of India for detailed assessment of autism and is mandated for evaluating degree of disability. It is a 40-item tool which can be used for certification and follow-up but **not** for screening [30]. ISAA was developed and validated in a multi-centric Pan-Indian study comprising 1124 participants aged 3-22 years with clinically diagnosed Autism/Pervasive Developmental Disorder (PDD-ICD10), Intellectual Disability (ID), other psychiatric disorders and persons with normal intellect. The scale was compared with Childhood Autism Rating Scale (CARS) (Version 1) [31]. Reliability and internal consistency of ISAA as compared to CARS was satisfactory [30, 31]. Validity of ISAA was determined through content validity and criterion validity. Initially an item pool of 437 items was generated, shortened to 57 after expert inputs from 30 experts, and then to 40 after a pilot study. At the end of the main study, each individual item was correlated with total ISAA scores which was significant at 0.001 level suggesting that all items were valid in differentiating between autism and other groups. The autism group had significantly higher scores than other groups suggesting high discriminant value. The criterion test validity of ISAA was determined by comparing total scores on ISAA with those on CARS using Pearson Product Moment Correlation (r=0.765, p<0.001). Internal consistency and reliability (Cronbach's coefficient alpha) were significant and comparable to CARS (Cronbach's alpha 0.932 P < 0.001) [31].However, the ISAA is a detailed evaluation tool which takes time and training, and cannot be used for population studies.

 4. We note that you have indicated that data from this study are available upon request. PLOS only allows data to be available upon request if there are legal or ethical restrictions on sharing data publicly. For information on unacceptable data access restrictions, please see [http://journals.plos.org/plosone/s/data-availability#loc-unacceptable-data-access-restrictions](http://journals.plos.org/plosone/s/data-availability#loc-unacceptable-data-access-restrictions).

*Reply 4: There are no ethical or legal restrictions. We have uploaded the data now.*

We will update your Data Availability statement on your behalf to reflect the information you provide.

 Query 5. Please amend the manuscript submission data (via Edit Submission) to include author Ravinder Singh.

*Reply 5: We apologize for this omission. We have added his name now.*

 Query 6. Please include a separate caption for each figure in your manuscript.

*Reply 6: We have added the separate captions.*

Query 7. Please include captions for your Supporting Information files at the end of your manuscript, and update any in-text citations to match accordingly. Please see our Supporting Information guidelines for more information: [http://journals.plos.org/plosone/s/supporting-information](about:blank)

*Reply 7: Captions for supporting information files are included.*

**Reviewers' comments:**

Reviewer's Responses to Questions

**Comments to the Author**

1. Is the manuscript technically sound, and do the data support the conclusions?

The manuscript must describe a technically sound piece of scientific research with data that supports the conclusions. Experiments must have been conducted rigorously, with appropriate controls, replication, and sample sizes. The conclusions must be drawn appropriately based on the data presented.

Reviewer #1: Yes

Reviewer #2: Yes

2. Has the statistical analysis been performed appropriately and rigorously?

Reviewer #1: Yes

Reviewer #2: Yes

3. Have the authors made all data underlying the findings in their manuscript fully available?

The [PLOS Data policy](http://www.plosone.org/static/policies.action#sharing) requires authors to make all data underlying the findings described in their manuscript fully available without restriction, with rare exception (please refer to the Data Availability Statement in the manuscript PDF file). The data should be provided as part of the manuscript or its supporting information, or deposited to a public repository. For example, in addition to summary statistics, the data points behind means, medians and variance measures should be available. If there are restrictions on publicly sharing data—e.g. participant privacy or use of data from a third party—those must be specified.

Reviewer #1: No

Reviewer #2: Yes

***Note: We have now uploaded all our study data.***

4. Is the manuscript presented in an intelligible fashion and written in standard English?

PLOS ONE does not copyedit accepted manuscripts, so the language in submitted articles must be clear, correct, and unambiguous. Any typographical or grammatical errors should be corrected at revision, so please note any specific errors here.

Reviewer #1: Yes

Reviewer #2: No

***Note: Thanks to our expert reviewers for their above remarks.***

**5. Review Comments to the Author**

Please use the space provided to explain your answers to the questions above. You may also include additional comments for the author, including concerns about dual publication, research ethics, or publication ethics. (Please upload your review as an attachment if it exceeds 20,000 characters)

Reviewer #1: Dear authors,
Thank you for the possibility to read your manuscript "Psychometric properties of a screening tool for autism in the community- the Indian Autism Screening Questionnaire (IASQ)". It describes an important area in autism spectrum disorders (ASD), and screening as an important factor in other countries and cultures.

*Reply: Thank you for your encouraging words. This is indeed an important first step regarding intervention planning for children with autism.*

The manuscript is describing the study in a good manner, however I have some comments;

Query 1- The manuscript states that "All children should be screened in the community at an early age". There are some scientific discussion on the efficacy of universal screening, and it is unclear what you base this statement on?

*Reply 1: We agree entirely with the expert reviewer, that universal early screening may identify false positives. However, it has significant advantages where healthcare services are less easily available. We have added the following text in introduction:*

Rashtriya Bal Swasthya Karyakram (RBSK) is a flagship Government of India Programme focused on early identification and intervention for children from birth to 18 years. It includes early identification of 4 ‘D’s viz. Defects at birth, Deficiencies, Diseases, Development delays including disability (including autism). [11]. For early identification all children should be screened in the community at a younger age. Community screening has several advantages [12].

Query 2: - The decision to use a binary scoring for each item is not described in the manuscript. As autism may be shown as qualitative differences in functioning, other studies has used a wider scale, and I wonder why this study did not?

*Reply 2: The study is aimed at screening the population by community health workers, who should be trained minimally. The screening instrument should be simple and easy to use by non-specialists. The diagnostic instrument ISAA uses wider scale as it should be used by expert trained workers. We have added the text in introduction for binary scoring as follows:*

We extracted these items and prepared a ten-item screening questionnaire with binary answers, because the binary answer format outperforms the popular seven-point multi-category format with respect to stability, concurrent validity, and speed of completion [34]. The tool needed to be simple enough to be scored by minimally trained workers.

Query 3: - The use of a cut-off score of 1 (out of 10), may pose risk for a high portion of false positives - even if this did not in this selected sample. This risk and possible consequences and remediation may be discussed to give the reader a better understanding

*Reply 3: We agree with our respected expert reviewer that cut off score of 1 may increase false positives but after screening, children need to be referred to a higher diagnostic centre and final diagnosis be made after expert review. In future, depending on the requirements, authors could use a different cut off. We have added a line in our revision thus:*

The overarching aim of this project was to design a screening instrument which would not miss even one child with autism when used in the community. Moreover, even those participants who were ‘false positives’ were not typical but had received a diagnosis of some other childhood psychiatric disorder which could be diagnosed at the second stage of detailed expert evaluation after screening. Out of a total of 145, only 4 participants were misdiagnosed as IASQ positive (clinically, one participant was diagnosed as Specific Developmental Disorder, 2 were diagnosed as Intellectual Disability and one was diagnosed as a behavior problem). Thus, for community screening, a cutoff score of 1 was decided by consensus. However, if a research study needs still higher specificity, a higher cut off may need to be used.

**Reviewer #2**: There were few grammatical errors in the abstract and in the introduction (ex. line 117 add "is"; line 118 add "a"). Perhaps also use "individuals" instead of "persons", or older children?

*Reply1: Thank you for pointing this out. We have changed the grammar accordingly.*

Query 2: one more thing to consider: the article is very nicely done. However, the population studied is relatively small. Perhaps mentioning this as a limitation as well, so to generalize in larger groups.

*Reply2: Thank you for appreciation. We have added the sample size issue as a limitation. Though our sample size was adequate for younger children, we needed a larger sample for older individuals. We have added the following text in limitations:*

Although we had recruited adequate sample (at least 10 participants per item) there were relatively fewer older individuals. In subsequent studies a larger sample for this group should be included to make this instrument more applicable to people of all ages.
